# Viscoelastic properties of small bowel mesentery at MR elastography in Crohn’s disease: a prospective cross-sectional exploratory study

**DOI:** 10.1186/s41747-023-00366-5

**Published:** 2023-09-18

**Authors:** Anne-Sophie van Schelt, Kim Johanna Beek, Nienke Petronella Maria Wassenaar, Eric M. Schrauben, Jurgen H. Runge, Krisztina Barbara Gecse, Jarmila D. W. van der Bilt, E. Andra Neefjes-Borst, Christianne Johanna Buskens, Aart J. Nederveen, Jaap Stoker

**Affiliations:** 1 Department of Radiology and Nuclear Medicine, Amsterdam UMC, University of Amsterdam, Amsterdam, The Netherlands; 2 Cancer Center Amsterdam, Imaging and Biomarkers, Amsterdam, The Netherlands; 3 Amsterdam Gastroenterology, Endocrinology, Metabolism, Amsterdam, The Netherlands; 4 Department of Radiology, Netherlands Cancer Institute, Amsterdam, The Netherlands; 5 Department of Gastroenterology and Hepatology, Amsterdam UMC, University of Amsterdam, Amsterdam, The Netherlands; 6 Department of Surgery, Amsterdam UMC, University of Amsterdam, Amsterdam, The Netherlands; 7 Department of Pathology, Amsterdam UMC, University of Amsterdam, Amsterdam, The Netherlands

**Keywords:** Crohn’s disease, Elasticity imaging techniques, Magnetic resonance elastography, Magnetic resonance imaging, Mesentery

## Abstract

**Background:**

Creeping fat is a pathological feature of small bowel Crohn’s disease (CD), with literature suggesting that bowel resection with extended mesenteric resection is related to less postoperative recurrences. Conventional imaging is unable to accurately quantify the disease involvement (*i.e.*, fibrosis) of creeping fat. Quantification of disease involvement could be useful in decision-making for additional extended mesenteric resection. We investigated the feasibility of magnetic resonance elastography (MRE) of the mesentery and if MRE is capable to detect fibrotic disease involvement of mesentery in active CD.

**Methods:**

Multifrequency MRE yielded spatial stiffness (shear wave speed, SWS, |G*|) and fluidity maps (φ). Viscoelastic properties of seven CD patients’ mesentery were compared to age- and sex-matched healthy volunteers (HV) (Mann–Whitney *U*-test). Within CD patients, the affected and “presumably” unaffected mesentery were compared (Wilcoxon-signed rank test). Repeatability was tested in 15 HVs (Bland–Altman analysis, coefficient of variation [CoV]). Spearman rank correlations were used to investigate the relation between microscopically scored amount of mesenteric fibrosis and viscoelastic parameters.

**Results:**

SWS, |G*|, and *φ* of affected mesentery in CD were higher compared to HV (*p* = 0.017, *p* = 0.001, *p* = 0.017). Strong correlations were found between percentage of area of mesenteric fibrosis and SWS and |G*| (*p* < 0.010). No differences were found within CD between affected and presumably unaffected mesentery. Repeatability of SWS showed 95% limits of agreement of (-0.09, 0.13 m/s) and within-subject CoV of 5.3%.

**Conclusion:**

MRE may have the potential to measure fibrotic disease involvement of the mesentery in CD, possibly guiding clinical decision-making with respect to extended mesenteric resection.

**Trial registration:**

Dutch trial register, NL9105, registered 7 December 2020.

**Relevance statement:**

MRE may have the potential to measure the amount of mesenteric fibrosis of the affected mesenteric fat in active Crohn’s disease, giving more insight into disease progression and could potentially play a role in clinical decision-making for extended mesenteric resection.

**Key points:**

• MRE of the mesentery in patients with active CD is feasible.

• Fluidity and stiffness of the mesentery increase in active CD, while stiffness correlates with the histopathological amount of mesenteric fibrosis.

• MRE provides biomarkers to quantify mesenteric disease activity in active CD.

**Graphical Abstract:**

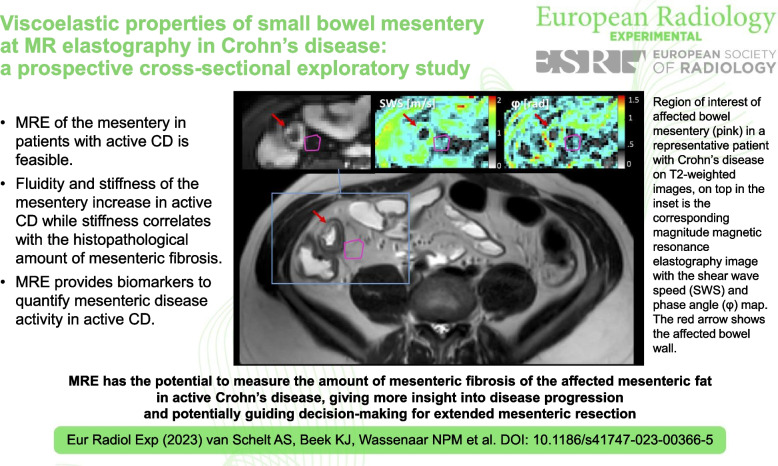

**Supplementary Information:**

The online version contains supplementary material available at 10.1186/s41747-023-00366-5.

## Background

Creeping fat is a pathological feature of small bowel Crohn’s disease (CD) at cross-sectional imaging and histopathology. It constitutes thickened, hypertrophic mesenteric adipose tissue (hereafter: mesentery) that wraps around the affected bowel wall. The exact mechanism of creeping fat is still debated and has been hypothesized as follows: (1) a passive and protective response to a dysfunctional gut barrier [1] and (2) an active part in the disease course with adipocytes secreting cytokines [2].

The presence of creeping fat at imaging is not an innocuous finding as it is associated with fibrosis and stricture formation of the affected bowel [3–6]. Currently, new surgical techniques are being studied to reduce postoperative recurrences. These techniques involve the mesentery either directly or indirectly (such as bowel resection with extended mesenteric resection or mesenteric exclusion by means of an anti-mesenteric Kono-S handsewn side-to-side anastomosis), and both techniques show less postoperative recurrences [7–10]. Current surgical guidelines [11] advise performing a mesenteric-sparing resection due to the benign character of CD. Extended mesenteric resection is not without risks and could result in a more extensive bowel resection as the latter vascularization is derived from the mesentery. Hence, critical thinking is imperative when extended mesenteric resection is considered. Characterizing the degree of mesenteric disease involvement (*i.e.*, fibrosis) could facilitate this decision-making.

Several studies have tried to quantify creeping fat either directly [12, 13] or indirectly [14–17]. A higher mesenteric fat index, an example of an indirect quantification, was shown to be correlated with an increased risk of early postoperative endoscopic recurrence of CD [15–17]. Recently, Li et al. [18] developed a novel mesenteric creeping fat index (MCFI) to quantify the amount of creeping fat. This was based on the extent of fat around the bowel circumference encompassed by vessels in the fat on computed tomography and showed an excellent correlation with the extent of fatty wrapping in resection specimens. Although promising, these methods do not provide information about the disease involvement (*i.e.*, fibrosis) of the mesentery.

Literature shows that creeping fat is histologically characterized by an increase in connective tissue (*i.e.*, fibrosis) and adipocyte hyperplasia [7]. Since an increase in connective tissue would lead to an increase in tissue stiffness, more advanced imaging techniques like elastography could play a role in identifying the amount of fibrosis. Lo Re et al. [19] supported this in a pilot study with ultrasound elastography in which fat around the affected bowel wall was stiffer compared to the fat around unaffected bowel wall in CD patients. Although promising, ultrasound elastography has some technical limitations such as a low penetration depth, dependence on transducer placement, and mapping is only performed in two dimensions [20].

On the contrary, magnetic resonance elastography (MRE) is a noninvasive technique that is able to quantify viscoelastic properties of soft tissue. This technique uses external mechanical vibrations [21] and measures the shear wave propagation throughout the entire abdomen. Using multiple transducers, at multiple frequencies, a three-dimensional overview of the local soft tissue stiffness in the abdomen is obtained. The research conducted with MRE to date has predominantly centered on diseases affecting the liver, such as liver fibrosis, cirrhosis, and hepatitis, as well as some other abdominal organs (*e.g.*, pancreas), certain brain, and musculoskeletal disorders [22]. MRE has been found to be particularly useful in assessing the severity of liver fibrosis, a condition in which there is excessive scarring of liver tissue that can lead to liver damage and dysfunction, as it can accurately measure the stiffness of liver tissue, which is a key indicator of fibrosis progression [23].

The aim of this study was to study the feasibility of mesenteric MRE and explore if this technique is able to detect mesenteric disease involvement (*i.e.*, fibrosis) in small bowel mesentery of active CD patients.

## Methods

### Study design

This prospective exploratory study was performed at a tertiary referral center (Amsterdam University Medical Center, location Academic Medical Center). Patients were recruited simultaneously with an overarching study (Dutch Trial Register NL9105; https://trialsearch.who.int/Trial2.aspx?TrialID=NL9105) from July 2021 until March 2022 and provided written informed consent. The study was conducted in agreement with the Declaration of Helsinki and was approved by the Medical Ethics Committee of the Amsterdam University Medical Center, location Academic Medical Center). Healthy volunteers signed informed consent and were recruited for protocol optimization with approval of the local Medical Ethics Committee by means of a waiver.

### Patients and healthy controls

Patients were eligible when they had endoscopic or histological-confirmed CD. Furthermore, patients were eligible when there was active CD of the small bowel and were scheduled for small bowel segment resection or had one or more small bowel stricture(s) (confirmed on endoscopy or cross-sectional imaging) that were scheduled for treatment with anti-inflammatory therapy. Patients were excluded if they had an isolated colonic stricture, were under 18 years of age, were unable to give informed consent, or if they had general MR imaging contraindications. Patient data consisted of sex, age, body mass index, Harvey-Bradshaw index, smoking status, disease duration in years, disease location and behavior according to the Montreal classification, type of previous surgery, and treatment given.

Fifteen healthy volunteers aged 18 years and older were recruited for repeatability analysis, which included seven age-sex-matched healthy controls (± 5 years). Healthy volunteers were excluded if they had a disease history of bowel disease (such as inflammatory bowel disease, celiac disease, diverticulitis, and a history of large or small bowel cancer) or had previous small or large bowel surgery. From the healthy volunteers, we collected disease history, sex, age, body mass index (kg/m^2^), and smoking status.

### Magnetic resonance protocol

**Fig. 1:**
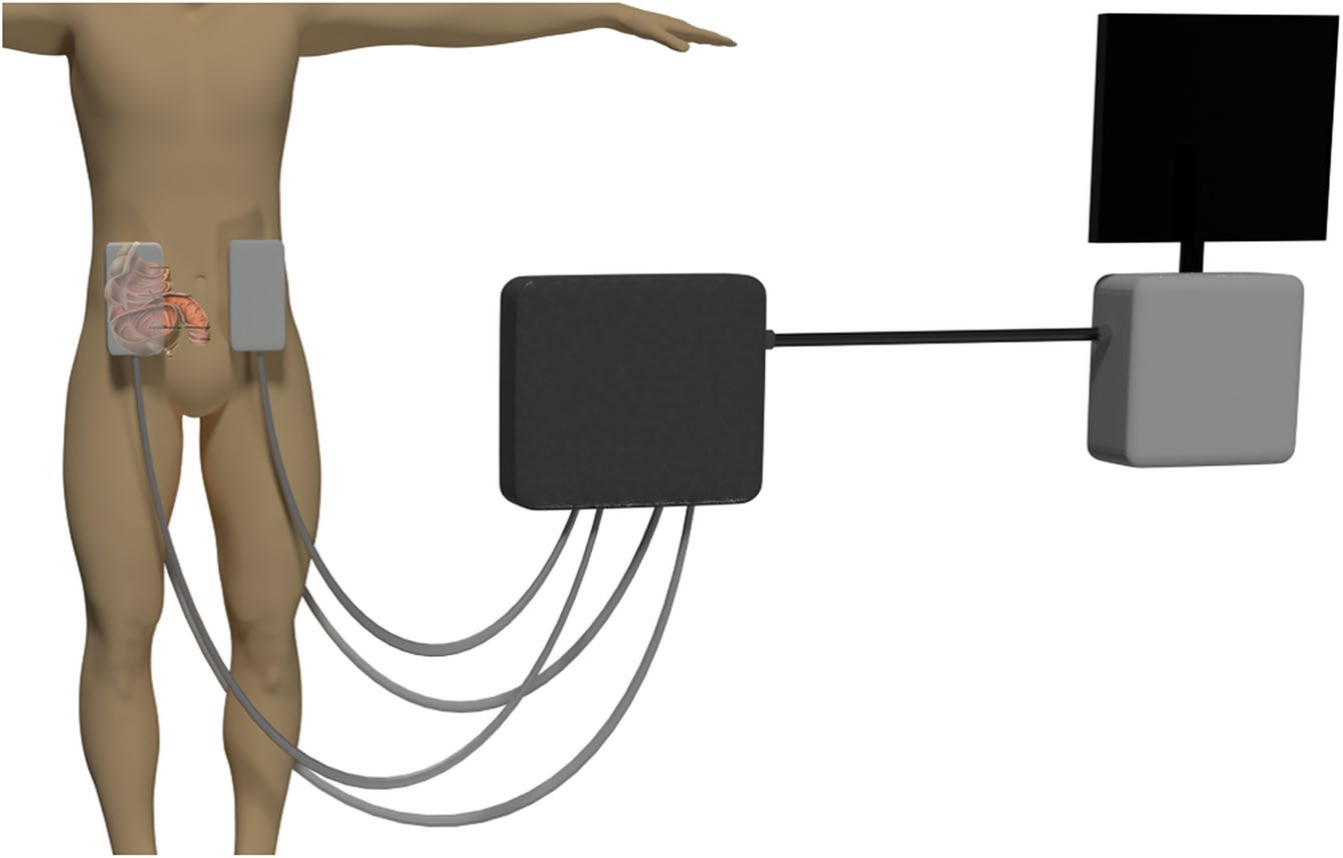
Research setup for magnetic resonance elastography of the small bowel mesentery. Four transducers are placed at the height of the ileocecal junction, two anterior and two posterior, and held in place by an elastic strap. The transducers are connected to a pressure box that controls the airflow to create a vibration

All scanning was performed on a 3 T scanner (Ingenia, Philips, Best, the Netherlands). All subjects fasted 4 h before scanning. Patients drank 1,600 mL of 1.9%-mannitol solution (Baxter B. V., Utrecht, the Netherlands) as an intraluminal contrast agent within 1 h before scanning as part of their routine MR enterography examination. Healthy volunteers drank 500 mL of water 15 min before scanning (mannitol was not allowed in healthy volunteers based on the protocol optimization waiver). MRE scans were performed using four pneumatic drivers placed at the height of the ileocecal junction (McBurney’s point was used as a derivate for the ileocecal junction), two anterior and two posterior. The four drivers were held in place with an elastic band (Fig. 1). A multifrequency spin-echo echo-planar imaging with spectral presaturation with inversion recovery MRE sequence was used (Table 1). For this sequence, free breathing was used with motion encoding gradients (MEGs) in three orthogonal directions to capture the full three-dimensional displacement field. Eight wave-phase offsets were measured, and mechanical frequencies ranged from 30 to 60 Hz with a 10-Hz increment. Furthermore, the magnetic resonance protocol for all participants consisted of T2-weighted images in coronal and axial orientation for anatomical reference. Repeatability was tested by performing two consecutive MRE acquisitions without repositioning, to test the within-session repeatability and ensure the most consistency in the location of the mesentery between both scans.

**Table 1:** MR parameters of the multifrequency MRE scan

Frequencies (Hz)	30, 40, 50, 60	Field of view (mm)	320 × 280 × 145
MEG duration (ms)	26.8, 26.8, 26.6, 22.2	Voxel size (mm^3^)	2.5 × 2.5 × 5
SENSE factor	2.5	Number of slices	29
TR/TE (ms)	2,400/55	Fold over direction	Anterior–posterior
MEG strength (mT/m)	24	Scan duration (min)	4

*MEG* Motion encoding gradient per frequency, *MRE* Magnetic resonance elastography, *SENSE* SENSitivity encoding, *TE* Echo time, *TR* Repetition time

### Post-processing

Post-processing of the MRE data was performed using the multifrequency dual elasto-visco (MDEV) and k-MDEV reconstruction algorithms [24, 25]. A Gaussian smoothing filter was applied before unwrapping the phase data for denoising. Unwrapping was done using a gradient method and a Laplacian filter for the MDEV and k-MDEV, respectively. A high-pass filter was used to filter the longitudinal waves; for the MDEV, an additional low-pass Butterworth filter was used. Taking the mean for all frequencies resulted in the mean shear stiffness (mean |G*|) in kilopascal (kPa) and phase angle (*φ*) in radians (rad) for the MDEV and the shear wave speed (SWS) in meters/seconds (m/s) for the k-MDEV. Furthermore, the octahedral shear strain signal-to-noise ratio (OSS-SNR) was calculated as a measure for MRE quality [21].

### Viscoelastic properties

Stiffness in tissue was determined by the SWS or |G*|, with higher SWS and |G*| values reflecting a higher tissue stiffness. Phase angle (*φ*) is a measure of tissue fluidity, *φ* > π⁄4 indicates predominantly fluid properties, while *φ* < π⁄4 indicates predominantly solid behavior [21]. An increase in the amount of water present in tissue increases fluidity and can be described as a viscous fluid [26].

### Segmentation

Segmentation was done manually by drawing a region of interest (ROI) on the MRE magnitude data with guidance of axial and coronal T2-weighted images. All segmentations were performed in ITK snap (version 3.4.0, Nov 30, 2015). Each ROI was placed in the mesentery close to the targeted small bowel loop, using the feeding mesenteric vessels to identify the proper mesentery. In CD patients, ROIs were placed in the mesentery of the most severely affected small bowel loop, defined as the part with the thickest bowel wall (> 3 mm) and smallest luminal diameter (≥ 50% luminal reduction compared to an unaffected bowel loop). In CD patients a ROI was also placed in the mesentery of healthy upstream small bowel at least 25 cm proximal to the diseased small bowel. Segmentations were supervised by an abdominal radiologist with 28 years of experience in gastrointestinal MR imaging (J.S.).

### Histopathology

In the fresh resection specimen, the small bowel segment with the thickest bowel wall and the most luminal narrowing was identified by a clinician (K.B.) and marked with a pin. Thereafter, the fresh resection specimen was kept for at least 24 h in formalin. After fixation, the indicated bowel segments were sectioned, photographed, and processed using hematoxylin and eosin staining.

**Fig. 2:**
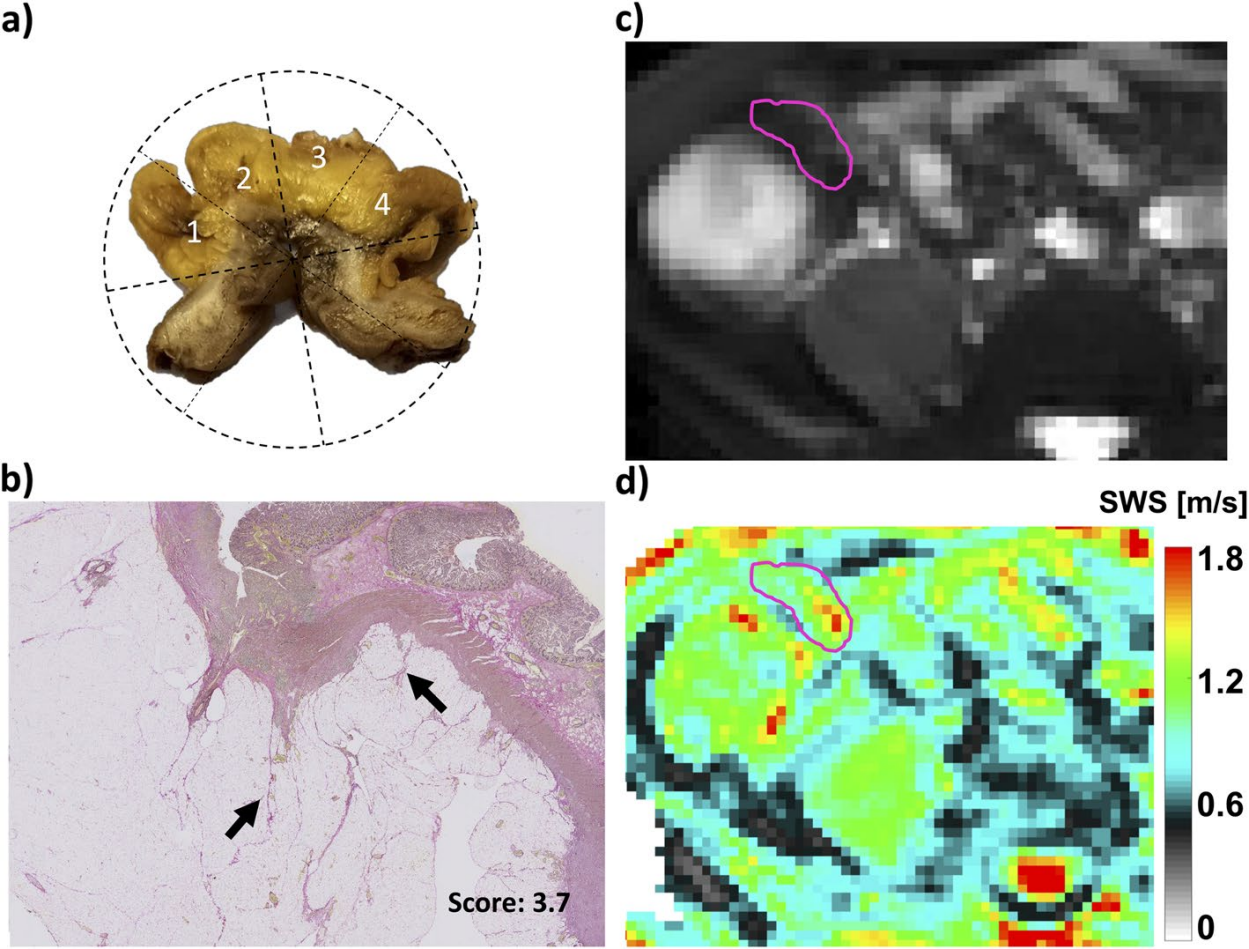
Examples are of a 21-year-old-male Crohn’s disease patient. **a** Macroscopic image of the resected small bowel section. Scoring is based on eight segments; four segments (1–4) are involved as is demonstrated. **b** Histopathological analysis (Elastica van Gieson stain) of a segment of the affected mesentery, the black arrows indicate fibrotic tissue. **c** Magnitude magnetic resonance elastography image with the affected mesentery in pink with the corresponding shear wave speed (SWS) map visible on the corresponding elastogram in the lower right corner

The amount of fatty wrapping around the gut was macroscopically scored by means of the mesenteric creeping fat index (MCFI) based on photos made after formalin fixation. MCFI is based on the amount of fatty tissue surrounding the bowel wall divided into eight clockwise segments (see Fig. 2a). The scoring was performed independently by two study coordinators (A.S. and K.B.). Afterwards, discrepancies were discussed, and the definitive MCFI scores per segment were determined, supervised by a pathologist specialized in gastrointestinal diseases with 31 years of experience (A.N.B.).

In order to quantitatively measure mesenteric fibrosis, the pathologist determined one section per patient which contained the most affected mesentery and delineated the affected area of interest. After digitalization of the sections, the area of interest was scored for fibrotic severity. This was done using an in-house built semiautomatic MATLAB software (v2021a, the MathWorks, Natick, MA, USA) that identified fibrosis within this area. Fibrosis was identified by a watershed segmentation technique, which was checked manually. The fibrotic severity was determined by the percentage of fibrosis with respect to the previously total delineated affected area, which yielded the microscopically scored amount of mesenteric fibrosis, denoted as the mesenteric fibrotic score in % (Fig. 2b).

### Statistical analysis

Continuous variables were presented as mean ± standard deviation or median with interquartile range (IQR) when skewed. Because of the small groups, we tested for normality with the Shapiro–Wilk test. Categorical variables were presented in proportions and percentages.

Average MRE parameters were determined by taking the mean with standard deviation over the ROI. Differences in viscoelastic properties for mesentery between active CD patients and healthy volunteers were analyzed by means of an unpaired *t*-test or Mann–Whitney *U*-test in case of skewed data. For differences in viscoelastic properties within CD patients, a paired *t*-test or Wilcoxon-signed rank test was used. Correlation analysis was done using the Pearson correlation coefficient or Spearman’s rho in case of non-normality.

Repeatability of MRE findings was analyzed by means of a Bland–Altman analysis resulting in the limits of agreement (LoA). Within-subject coefficients of variation (CoV) were determined for all MRE. Values of *p* lower than 0.05 were considered significant in all other assessments. No power calculation was performed because of the exploratory nature of this study.

## Results

### Study population

Our case–control population (Table 2) consisted of seven patients (median age 39 years; 5 males) with active stricturing small bowel CD and seven healthy volunteers (median age 34 years; 5 males), without a difference in age (*p* = 0.902). All patients had either stricturing or penetrating disease according to the Montreal classification [27]. On conventional magnetic resonance imaging, all patients had a small bowel stricture (bowel wall thickness > 3 mm and luminal narrowing ≥ 50% compared to normal bowel loop). The patients had a relatively long disease duration of 7 years (IQR 2–12). Six of the seven patients underwent ileocecal resection (*n* = 5) or ileocecal re-resection (*n* = 1) within median of 39 days (IQR 23–61) after MRE acquisition. One patient started with anti-inflammatory treatment. The repeatability study population consisted of 15 healthy volunteers, aged 31 ± 7 years (mean ± standard deviation), and 6 males. All scans were of sufficient quality for analysis.

**Table 2:** Baseline characteristics of both patients with active Crohn’s disease (CD) as sex- and age-matched healthy controls

Characteristics in median [IQR], mean (± SD) or *n* (%)	Patients with active CD (*n* = 7)	Healthy volunteers (*n* = 7)	*p*-value
Sex (*n*, % male)	5 (71.4%)	5 (71.4%)	
Age (years)	39 [23–54]	34 [26–55]	0.902
Body mass index (kg/m^2^)	25.3 [17.9–28.9]	25.7 [24.6–26.8]	0.902
Harvey-Bradshaw index	7.1 (± 3.1)		
Smoking status			
*Never smoked*	4 (57.1%)	6 (85.7%)	
*Previous smoker*	2 (28.6%)	−	
*Current smoker*	1 (14.3%)	1 (14.3%)	
Disease duration (years)	7 [2–12]	−	
Disease location			
*L1 — ileal*	4 (57.1%)		
*L3 — ileocolonic*	3 (42.9%)		
Disease behavior			
*B1 —– nonstricturing-nonpenetrating*	−		
*B2 — stricturing*	5 (35.7%)		
*B3 — penetrating*	2 (14.3%)		
Type of previous surgery			
*Ileocecal resection*	2 (28.6%)		
Treatment			
*Ileocecal resection*	5 (71.3%)		
*Ileocecal reresection*	1 (14.3%)		
*Anti-inflammatory treatment*	1 (14.3%)		
Length between affected and unaffected bowel wall (cm)	47.0 (25.0–60.0)		

*IQR* Interquartile range, *SD* Standard deviation

### Viscoelastic parameters of the mesentery

**Fig. 3:**
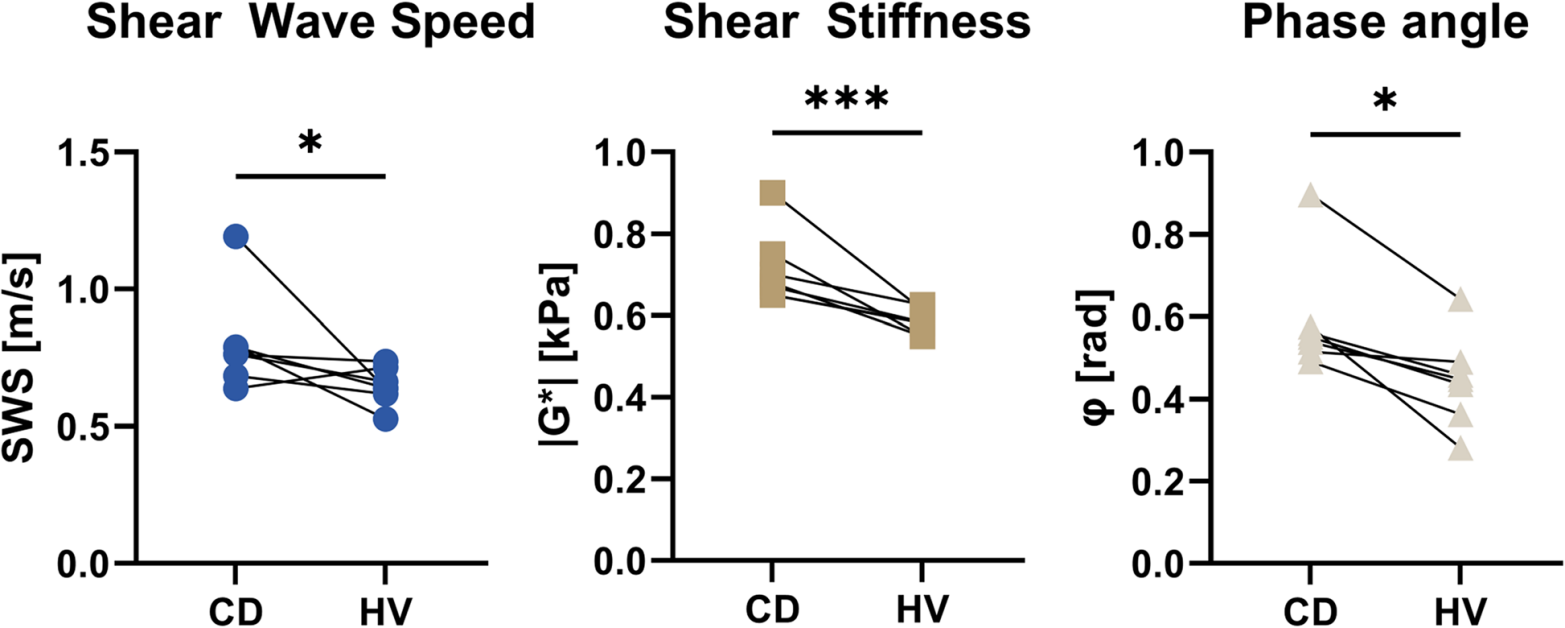
Comparison of magnetic resonance elastography (shear wave speed, shear stiffness, and phase angle) parameters of the most affected mesentery between Crohn’s disease patients (CD) and healthy volunteers (HV) at the height of the ileocecal junction (*p < 0.05, ***p < 0.001)

**Fig. 4:**
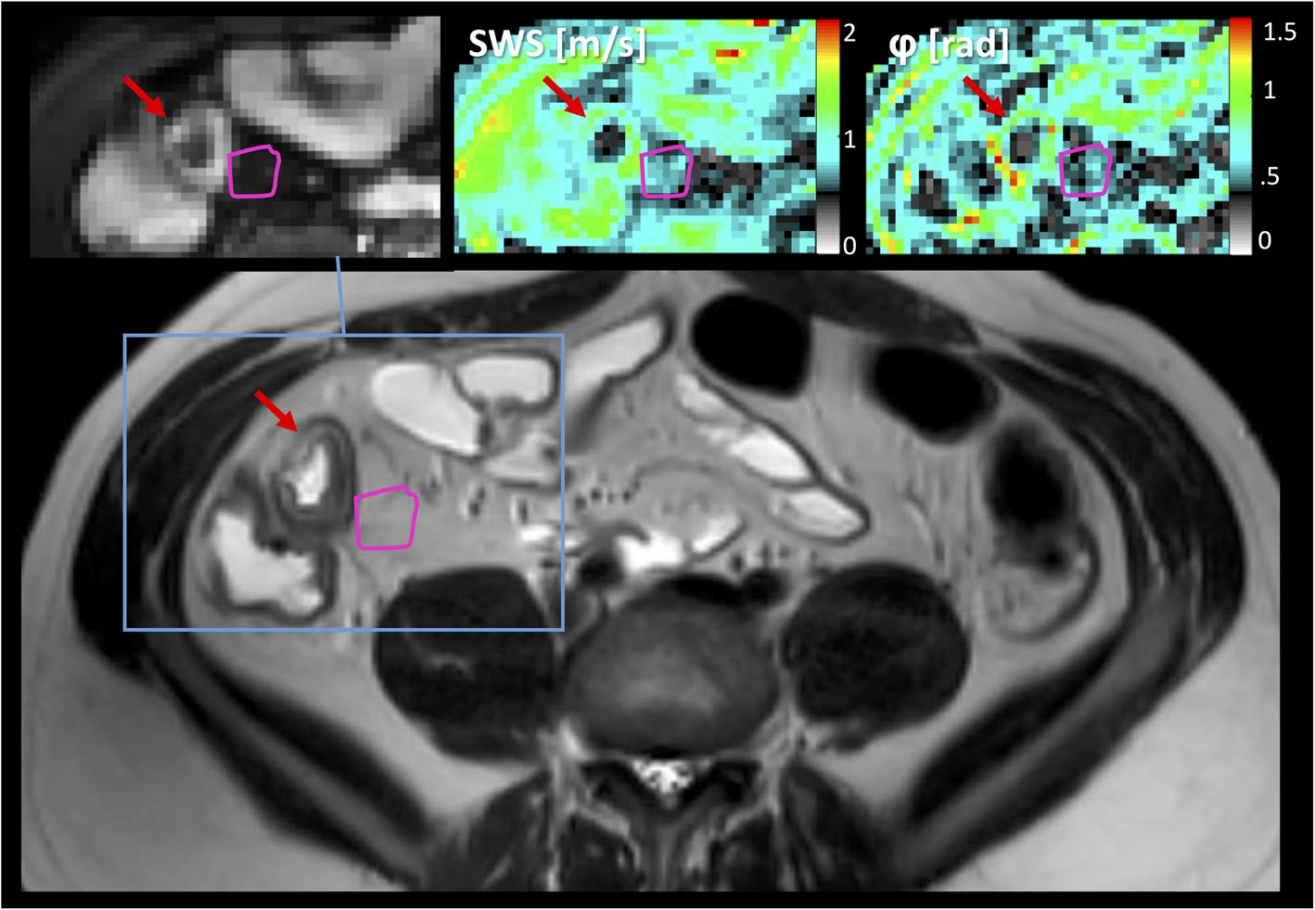
Region of interest of affected bowel mesentery in a representative patient with Crohn’s disease on T2-weighted images, on top in the inset is the corresponding magnitude magnetic resonance elastography image with the shear wave speed (SWS) and phase angle (φ) map. Segmented affected mesentery is highlighted in pink, and the red arrow shows the affected bowel wall

All ROIs had a minimal size of 31.25 mm^2^. SWS, |G*|, and *φ* of affected mesentery in CD patients were higher compared to the mesentery of healthy volunteers (*p* = 0.017, *p* = 0.001, and *p* = 0.017, respectively) (Fig. 3). SWS, |G*|, and *φ* between affected mesentery and “presumably” unaffected mesentery (at least 25 cm proximal to the diseased small bowel) within CD patients showed no significant differences (*p* = 0.318, *p* = 0.620, and *p* = 0.209, respectively). A representative patient is shown in Fig. 4. The median SWS of affected mesentery in CD patients was 0.76 m/s (IQR 0.68–0.79), while the median mesentery of healthy volunteers was 0.64 m/s (IQR 0.53–0.74). All viscoelastic parameters are reported in the supplementary material (Table S1). Mean OSS-SNR values for the healthy mesentery were 3.6 ± 1.8 dB, while the affected mesentery in CD patients had a OSS-SNR of 5.5 ± 1.4 dB (*p* = 0.123).

### Histopathology of the mesentery

**Fig. 5:**
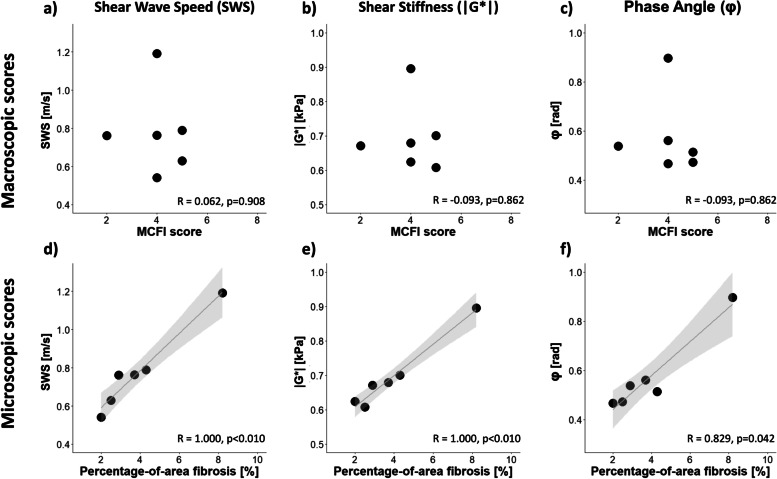
Macroscopic scoring. **a** Shear wave speed (SWS), (**b**) shear stiffness (|G*|), and (**c**) phase angle (φ) as a function of mesenteric creeping fat index (MCFI). Microscopic scoring: (**d**) SWS, (**e**) |G*|, and (**f**) (φ) as a function of mesenteric fibrosis score (percentage of area of fibrosis (%) within a region of interest). Spearman’s rho and corresponding *p*-values are given

Histopathological analysis was done in six patients who underwent ileocecal (re-)resection. The mean macroscopic MCFI score based on six resection specimens was 4 ± 1. No correlation was found between macroscopic MCFI score and viscoelastic parameters: (*r*(4) = 0.062, *p* = 0.908 for SWS and |G*|; *r*(4) = -0.555, *p* = 0.252 for *φ* (Fig. 5a–c). A strong positive correlation between mesenteric fibrotic score for both SWS (*r*(4) = 1.000, *p* < 0.010) and |G*| (*r*(4) = 1.000, *p* < 0.010) was found, but not for *φ* (*r*(4) = 0.429, *p* = 0.397). Macro- and microscopic scores as a function of viscoelastic parameters are shown in Fig. 5d–f. The mesenteric fibrotic score for all patients was 3.9 ± 2.2% (mean ± standard deviation). Figure 2 depicts an example of an elastogram of a 21-year-old male with active CD with histopathological analysis after ileocecal resection.

### Repeatability

**Fig. 6:**
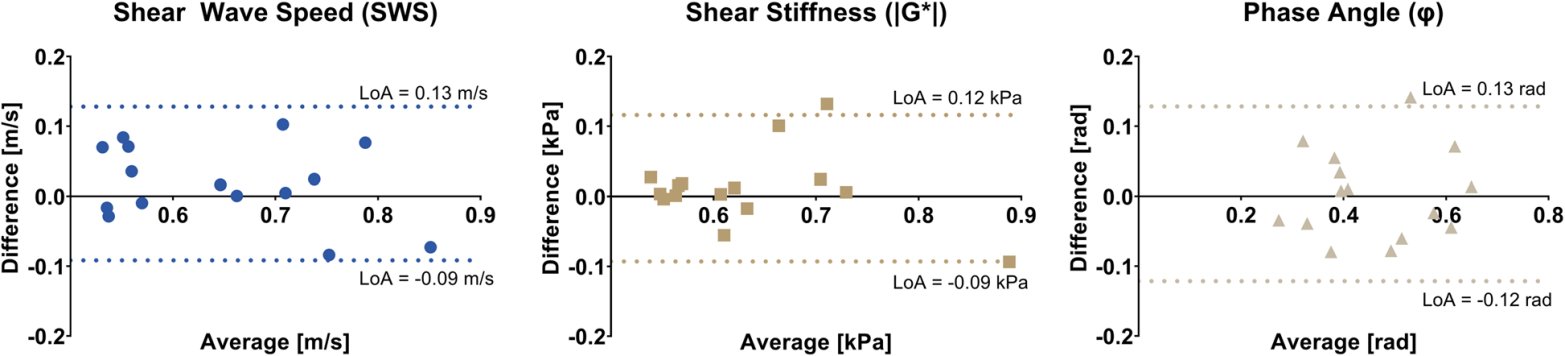
Bland–Altman analysis of the magnetic resonance elastography repeatability of healthy volunteers. The 95% limits of agreement (LoA) and bias are given per viscoelastic parameter: shear wave speed (SWS), shear stiffness (|G*|), and phase angle (φ)

In the repeatability population (*n* = 15), mean SWS, |G*|, and *φ* were 0.64 ± 0.11 m/s, 0.63 ± 0.10 kPa, and 0.46 ± 0.12 rad (mean ± standard deviation), respectively. LoA were [-0.09, 0.13 m/s], [-0.09, 0.12 kPa], and [-0.12, 0.13 rad], respectively. Within-subject CoV were 8.7%, 8.4%, and 14.0%, respectively. Bland–Altman analysis of the repeatability in healthy volunteers is shown in Fig. 6.

## Discussion

We demonstrated that MRE of the small bowel mesentery can show differences in viscoelastic properties between active CD patients and age- and sex-matched healthy volunteers. The increase in SWS and |G*| in the mesentery of CD patients indicates an increased stiffness, while the increased *φ* indicates increased fluidity. To our knowledge, this is the first study to look into viscoelastic properties of small bowel mesentery with MRE in patients with active CD and corresponding histopathology.

Increased stiffness suggests an increase in connective tissue present in the mesentery in active CD patients. This is also supported by recent literature which demonstrated that 88% of CD patients with stricturing or penetrating CD (according to the Montreal classification) had penetrating fibrosis at histopathology defined as fibrosis that was continuous through all intestinal layers with extension into the mesenteric fat [28, 29].

The amount of mesenteric fibrosis at histopathology (mesenteric fibrotic score) showed a strong positive linear relation with stiffness (SWS) and shear stiffness (|G*|) measured by means of MRE within active CD patients. This suggests that measuring the SWS and |G*| with MRE could be used to determine the amount of mesenteric fibrosis in patients with active CD. This also suggests that MRE could potentially be used as a biomarker, which gives insight into the degree of mesenteric disease involvement (*i.e.*, fibrosis).

For both the CD patients and healthy volunteers, *φ* was below *π*/4, meaning the mesentery presents as an elastic-solid. The increased fluidity can indicate the presence of disorganized proteins, vascularity, or increased amount of adipocytes, which increases pressure and mechanical friction [29–31]. However, this has not been explored yet; further research in fluidity is necessary to corroborate this.

Previous literature states that 50% is the minimal amount of fatty wrapping for the definition of creeping fat [7]. The MCFI showed an average value of 4, which implies at least 50% of the bowel surface of the resected specimens was covered with fatty tissue and therefore creeping fat. Nonetheless, the MCFI showed no correlation with any mesenteric viscoelastic parameter and suggests that mesenteric viscoelastic properties have no interdependence with creeping fat. However, the cohort is too small to corroborate hard conclusions based on this finding.

In regards to the comparison within CD patients between affected and presumably unaffected mesentery, no significant differences were found for each of the viscoelastic parameters. One can speculate based on these findings that mesenteric involvement in active CD may be more extensive than seen at conventional imaging. This is supported by Büning et al. [32] who showed that patients with stricturing CD had a higher visceral adipose tissue/total fat mass ratio and proinflammatory cytokines (interleukin-6) compared to patients with nonstricturing CD. Likewise, the study showed that there were more patients with higher visceral adipose tissue/total fat mass ratios in short-term remission than in long-term remission.

Our study displays a reliable repeatability of MRE of the mesentery in healthy volunteers. The LoA and CoV are in line with literature that has used similar methods in other abdominal applications [33–35]. Furthermore, the measured difference between CD patients and healthy volunteers surpasses the within-subject CoV, meaning that the measured differences are greater than the acquisition variability. However, it is important to note that no repositioning took place between MRE scans. This was to ensure the most optimal overlap in the ROI between both scans of the same part of the mesentery. This approach was considered more crucial in a feasibility study with histopathology correlation, although it may have affected the repeatability outcome by potentially inflating it. Several limitations exist in our study.

First, we performed an exploratory study with a small patient cohort. This precludes firm conclusions, although the findings suggest a role of MRE in determining the amount of mesenteric disease involvement (*i.e.*, fibrosis) in active CD patients. Moreover, the spin-echo echo-planar imaging sequence requires fat suppression to reduce EPI artifacts due to high fat signal. Smaller fat signals in proximity to the main water peak are not suppressible with spectral presaturation with inversion recovery yet cause a minimal water-fat shift. The remaining signal is lower, but is not zero as a result of incomplete fat suppression. Nonetheless, the OSS-SNR in subjects was of sufficient amount to determine the viscoelastic properties and notably larger in affected mesentery in CD patients. This could be due to infiltration of other tissues (*i.e.*, fibrosis) in the mesenteric fat. Therefore, differences in viscoelastic properties between active CD patients and healthy volunteers are caused by differences in underlying tissue structures.

Furthermore, our patient population all had long-standing Crohn’s disease, and they underwent surgical resection as this study was designed to include histopathological verification. Therefore, this patient cohort does not reflect the entire CD patient population. It would be interesting to investigate mesenteric viscoelastic properties in patients with a shorter disease duration. If the mesenteric viscoelastic properties between patients with long-standing disease differ between patients with a shorter disease duration, this could possibly play a role in clinical decision-making with respect to either surgical or medical treatment.

To conclude, our findings suggest that MRE may have the potential to measure the amount of mesenteric fibrosis of the affected mesenteric fat in active CD. Therefore, it could give more insight into disease progression and the role of the mesentery in the disease course. Future research should look into the value of MR elastography concerning viscoelastic properties of small bowel mesentery in a more heterogeneous group of patients with CD, with advanced and less advanced disease progression. Moreover, the quantitative nature of MRE allows for the exploration of patient’s stratification options, based on the amount of mesenteric fibrosis. Defining cutoff values for mesenteric fibrosis detection could help surgeons in their decision-making whether to perform an extended mesenteric resection or conventional bowel surgery. This could contribute to a proper identification of the added value MRE could play in managing patients with active CD.

### Supplementary Information

**Additional file 1: Table S1.** Comparison of viscoelastic properties of affected small bowel mesentery in Crohn’s Disease patients and mesentery in healthy volunteers, and between affected and ‘presumably’ unaffected mesentery within Crohn’s Disease patients (*p<0.05, ***p ≤0.001).

## Data Availability

The datasets used and analyzed during the current study are available from the corresponding author on reasonable request.

## Abbreviations

|G*|: Shear stiffness
3D: Three dimensional
CD: Crohn’s disease
CoV: Coefficient of variation
IQR: Interquartile range
LoA: Limits of agreement
MCFI: Mesenteric creeping fat index
MDEV: Multifrequency dual elasto-visco
MEGs: Motion encoding gradients
MRE: Magnetic resonance elastography
OSS-SNR: Octahedral shear strain signal-to-noise ratio
ROI: Region of interest
SWS: Shear wave speed
