# Supplementary material for: Viscoelastic properties of small bowel mesentery at MR elastography in Crohn’s disease: a prospective cross-sectional exploratory study
Source: Eur Radiol Exp. 2023 Sep 18;7:53. doi: 10.1186/s41747-023-00366-5 (PMC10505604; doi:10.1186/s41747-023-00366-5)
Supplement: Supplementary file 1 — Additional file 1: Table S1. Comparison of viscoelastic properties of affected small bowel mesentery in Crohn’s Disease patients and mesentery in healthy volunteers, and between affected and ‘presumably’ unaffected mesentery within Crohn’s Disease patients (*p<0.05, ***p ≤0.001). [file 41747_2023_366_MOESM1_ESM.pdf]

Viscoelastic properties of small bowel mesentery at MR elastography in Crohn's disease: a prospective cross-sectional exploratory study

<ins>**ELECTRONIC SUPPLEMENTARY MATERIAL**</ins>

Table S1. *Comparison of viscoelastic properties of affected small bowel mesentery in Crohn's Disease patients and mesentery in healthy volunteers, and between affected and 'presumably' unaffected mesentery within Crohn's Disease patients (*p<0.05, ***p≤0.001)*

		**CD patients**		**HV**	**CD affected – HV mesentery**				**affected - unaffected mesentery**			
		Affected	Unaffected	Healthy Volunteers	U	z	*p*-value	r	U	z	*p*-value	r
Median [IQR]												
	**SWS [m/s]**	0.76[0.68-0.79]	0.68[0.63-0.81]	0.64[0.53-0.74]	43	2.4	0.017*	0.6	35	1.3	0.318	0.3
	**|G*| [kPa]**	0.68[0.65-0.75]	0.67[0.57-0.79]	0.58[0.55-0.63]	49	3.1	0.001***	0.8	34	1.2	0.620	0.3
	**φ [rad]**	0.55[0.51-0.58]	0.52[0.47-0.54]	0.45[0.28-0.64]	43	2.4	0.017*	0.6	12	-1.6	0.209	0.4
